# The nitrite reductase encoded by *nirBDs* in *Pseudomonas putida* Y-9 influences ammonium transformation

**DOI:** 10.3389/fmicb.2022.982674

**Published:** 2022-10-12

**Authors:** Xuejiao Huang, Yuwen Luo, Luo Luo, Deti Xie, Zhenlun Li

**Affiliations:** ^1^Key Laboratory of (Guangxi) Agricultural Environment and Products Safety, College of Agronomy, Guangxi University, Nanning, China; ^2^Chongqing Key Laboratory of Soil Multiscale Interfacial Process, Southwest University, Chongqing, China; ^3^Guangxi Bossco Environmental Protection Technology Co., Ltd., Nanning, China

**Keywords:** *Pseudomonas putida* Y-9, *nirBDs*, *glnA*, ammonium assimilation, ammonium oxidation

## Abstract

It is unknown whether *nirBDs*, which conventionally encode an NADH nitrite reductase, play other novel roles in nitrogen cycling. In this study, we explored the role of *nirBDs* in the nitrogen cycling of *Pseudomonas putida* Y-9. *nirBDs* had no effect on organic nitrogen transformation by strain Y-9. The △*nirBD* strain exhibited higher ammonium removal efficiency (90.7%) than the wild-type strain (76.1%; *P* < 0.05) and lower end gaseous nitrogen (N_2_O) production. Moreover, the expression of *glnA* (control of the ammonium assimilation) in the △*nirBD* strain was higher than that in the wild-type strain (*P* < 0.05) after being cultured in ammonium-containing medium. Furthermore, nitrite noticeably inhibited the ammonium elimination of the wild-type strain, with a corresponding removal rate decreasing to 44.8%. However, no similar impact on ammonium transformation was observed for the △*nirBD* strain, with removal efficiency reaching 97.5%. In conclusion, *nirBDs* in strain Y-9 decreased the ammonium assimilation and increased the ammonium oxidation to nitrous oxide.

## Introduction

Microorganisms show multiple nitrogen transformation pathways, contributing to the natural nitrogen-cycling balance (Canfield et al., 2010; Kuypers et al., 2018). Ammonium (NH_4_^+^) is the preferred nitrogen source for most bacteria and archaea (Burkovski, 2003; Muro-Pastor et al., 2005). Ammonium assimilation by microorganisms leads to high ammonium removal efficiency and mitigation of harmful effects on the environment. However, the role of ammonium assimilation in NH_4_^+^ removal is often neglected.

Simultaneous nitrification and denitrification (SND) has been widely applied in wastewater treatment plants as an attractive biological approach to nitrogen removal, owing to its low investment costs and high efficiency (Jin et al., 2015; Lei et al., 2016). Numerous studies have focused on isolating, identifying, and characterizing nitrogen removal associated with SND strains (Jin et al., 2015; Chen et al., 2016, 2021; Lei et al., 2019). Usually, the SND pathway by bacteria is NH_4_^+^→NH_2_OH→NO_2_^–^→NO_3_^–^→NO_2_^–^→NO→N_2_O →N_2_. Ammonia monooxygenase catalyzes NH_4_^+^ oxidization to NH_2_OH and is encoded by *amoA*, *amoB*, or *amoC* (Kuypers et al., 2018). Nitrate reductase encoded by *narG*, *narH, napA*, or *nasA* can catalyze NO_3_^–^ reduction to NO_2_^–^. Nitrite reductase catalyzing NO_2_^–^ reduction to NO or NH_4_^+^ is encoded by *nirK*, *nirS, nirB*, or *nirD* (Kuypers et al., 2018; Yang L. et al., 2019; Xia et al., 2020; Zhang et al., 2020).

*nirBDs*, which conventionally encode an NADH nitrite reductase in many bacteria (Jackson et al., 1981; Lin and Stewart, 1997; Malm et al., 2009), show different characteristics under different conditions. For example, dissimilatory nitrite reductase encoded by *nirBDs* in *Escherichia coli* and other enterobacteria is only expressed under anaerobic conditions (Macdonald et al., 1985; Gennis and Stewart, 1996), while *nirBDs* in *Streptomyces coelicolor* only encode the assimilatory nitrite reductase under aerobic conditions (Tiffert et al., 2008; Fischer et al., 2012). Recent studies have shown that *nirBDs* in *S. coelicolor* also play an integral role in the nitric oxide (NO) homeostatic regulation system that eliminates nitrite (NO_2_^–^) from cultures during NO_3_^–^ reduction (Yukioka et al., 2017).

We previously observed that the SND strain *Pseudomonas putida* Y-9 exhibited excellent removal ability for NH_4_^+^ and NO_3_^–^ (Xu et al., 2017). Further studies clarified that strain Y-9 could transform NH_4_^+^ into nitrous oxide (N_2_O) under aerobic conditions (Huang et al., 2019) and remove NH_4_^+^ mainly through assimilation (Huang et al., 2021a). In addition, strain Y-9 can remove NO_3_^–^ via simultaneous nitrate assimilation, dissimilatory nitrate reduction to ammonium (DNRA), and denitrification under aerobic conditions. Within these contexts, the enzyme encoded by *nirBDs* catalyzes NO_2_^–^ reduction to NH_4_^+^ during assimilation and DNRA (Huang et al., 2020). We hypothesized that *nirBDs* in strain Y-9 might be functional in other roles than encoding the traditional nitrite reductase. In this study, the effects of *nirBDs* on different nitrogen transformation pathways in strain Y-9 were explored. Results revealed that knocking out *nirBDs* promoted ammonium assimilation and weakened the emission of nitrous oxide. These findings provide a new understanding on how to use strain Y-9 for the treatment of ammonium nitrogen polluted water.

## Materials and methods

### Strain and culture media

The SND bacterium *P. putida* Y-9 (GenBank No. KP410740) used here was obtained from our previous study (Xu et al., 2017). *nirBDs* were knocked out from the genome of strain Y-9 using homologous recombination technology, as previously described (Huang et al., 2020), mediated via plasmid pLP12. The primer sequences for *nirBDs* knockout are shown in Supplementary Table 1. Supplementary Figure 1 shows the successful construction of the *nirBD* deletion mutants.

Lysogeny broth (LB) liquid medium consisting of (per liter) 10.0 g Tryptone, 5.00 g Yeast extract, and 10.0 g NaCl (pH adjusted to 7.0–7.2) was used for strain enrichment.

Nitrification medium (NM) comprised (per liter) 7.00 g K_2_HPO_4_, 3.00 g KH_2_PO_4_, 0.10 g MgSO_4_⋅7H_2_O, 0.50 g (NH_4_)_2_SO_4_, 0.05 g FeSO_4_⋅7H_2_O, and 5.13 g CH_3_COONa (pH adjusted to 7.2). NM was used to determine the ammonium transformation characteristics of strain Y-9.

The composition of the denitrification medium (DM) was (per liter) 7.00 g K_2_HPO_4_, 3.00 g KH_2_PO_4_, 0.10 g MgSO_4_⋅7H_2_O, 0.72 g KNO_3_ (DM-1) or 0.49 g NaNO_2_ (DM-2), 0.05 g FeSO_4_⋅7H_2_O, and 5.13 g CH_3_COONa (pH adjusted to 7.2). The two DM formulae were used to evaluate the nitrate or nitrite transformation ability of strain Y-9.

The organic nitrogen medium (OM) was composed of (per liter, pH 7.2) 7.00 g K_2_HPO_4_, 3.00 g KH_2_PO_4_, 0.10 g MgSO_4_⋅7H_2_O, 0.79 g tryptone, 0.05 g FeSO_4_⋅7H_2_O, and 0.788 g peptone (pH adjusted to 7.2). OM was used to evaluate the organic nitrogen conversion ability of strain Y-9.

The SND medium contained 7.00 g K_2_HPO_4_, 3.00 g KH_2_PO_4_, 0.10 g MgSO_4_⋅7H_2_O, 0.50 g (NH_4_)_2_SO_4_, 0.72 g KNO_3_ (SND-1) or 0.49 g NaNO_2_ (SND-2), 0.05 g FeSO_4_⋅7H_2_O, and 10.3 g CH_3_COONa (pH adjusted to 7.2). Two types of SND media were used to assess the nitrogen transformation ability of strain Y-9 with ammonium and either nitrate or nitrite.

Solid plates were prepared using 2.0% (w/v) agar added into the above liquid media. Before use, all of the above media were autoclaved for 30 min at 0.11 MPa and 121^°^C.

### Estimation of the role of *nirBDs* in nitrogen transformation by strain Y-9

Single colonies of the Y-9 and △*nirBD* strains were enriched for 36 h using LB liquid medium. Preculture (8 mL) was harvested and washed twice with sterile pure water by centrifugation (4,000 rpm, 8 min), inoculated into 100 mL of NM, DM-1, DM-2, OM, SND-1, or SND-2, and then cultivated at 15^°^C with shaking at 150 rpm. No strains were added for control treatments. Three replicates were performed for each experiment. Culture samples in different nitrogen media were taken out to measure the optical density of the strain (OD_600_) and different types of nitrogen using a spectrophotometer (Huang et al., 2019).

### Detection of N_2_O and N_2_ after Y-9 and △*nirBD* strains cultured in ammonium medium

The precultures of the Y-9 and the △*nirBD* strains were inoculated into media containing (^15^NH_4_)_2_SO_4_ (10 atom%) in 250 mL serum bottles. Then, the serum bottles were sealed with a rubber septum, aerated with oxygen, and incubated at 15°C for 48 h with shaking at 150 rpm. Finally, the ^15^N_2_O and ^15^N_2_ present in the headspace were collected with a needle and detected using GC-MS (Agilent, USA) and GC-IRMS (Thermo Fisher Scientific, USA), respectively (Ai et al., 2011; Ye et al., 2016; Huang et al., 2019).

### Monitoring the expression of *glnA*

DNA fragments of the Y-9 and △*nirBD* strains containing upstream regions of *glnA* were PCR-amplified and ligated into the upstream region of the promoter-less *lacZ* in pRG970Km (Table 1) to generate the reporter plasmids p970 Km-glnA. Then, the Y-9 and △*nirBD* strains containing the reporter plasmids were cultured in NM medium with shaking at 150 rpm, and aliquots were collected after 2 days of incubation. The activity of β-galactosidase was measured as described by Miller (1972). Relative expression of glutamine synthetase encoded by *glnA* was represented by OD_420_/OD_600_ (Xu et al., 2018). Moreover, total RNA of Y-9 and △*nirBD* strains incubated in NM medium for 48 h was extracted and converted to cDNA to investigate the relative expression of *glnA* using qPCR. The primers of *glnA* were GACCACGAAATCCGTACTGC and TTTCAGGGCCTGTACTTCGT. The 16S rRNA gene was used as an internal standard, and its primers were GTGCCAGCMGCCGCGG (515F) and CCGTCAATTCMTTTRAGTTT (907R). The PCR cycling conditions were as follows: initial denaturation at 95°C for 30 s; 38 cycles at 95°C for 15 s, 60°C for 30 s, and 72°C for 30 s; 1 cycle of 95°C for 15 s; and finally, stepwise temperature increases from 55 to 95°C to generate the melting curve. Standard curves were established using a dilution series of pMD19-T vectors containing the target gene (Huang et al., 2021b).

**TABLE 1:** Strains and plasmids used in this study.

Strain or plasmid	Description	References or source
**Strains**		
*Pseudomonas putida* Y-9	Wild type	Xu et al., 2017
△*nirBD* Y-9	*nirBD* genes in-frame deletion in strain Y-9; Km*^r^*	Huang et al., 2020
*E. coli*	*DH5*αλ*-ϕ80dlacZ*Δ*M15*Δ*(lacZYA-argF)U169 recA1 endA1 hsdR17(rK- mK-) supE44 thi-1 gyrA relA1*	Lab stock
**Plasmids**		
pRG970Km	Cloning vector containing promoterless *lacZYA* for construction of transcriptional fusion; Km*^r^*	Yan et al., 2009
p970Km-glnA	pRG970 Km containing a *glnA* transcriptional fusion; Km*^r^*	This study

### Analytical methods

Total nitrogen (TN, including cells) content in the suspension was estimated using the alkaline potassium persulfate digestion-UV spectrophotometric method. The contents of NH_4_^+^, NO_2_^–^, and NO_3_^–^ in the supernatant were quantified using the indophenol blue method, the hydrochloric acid photometry method, and the N-(1-naphthalene)-diaminoethane spectrophotometry method, respectively, after samples were centrifuged at 8,000 rpm for 5 min. The above analyses were carried out according to the guidelines set by the State Environmental Protection Administration of China (2002). The decline rate of nitrogen (TN, NH_4_^+^, NO_2_^–^, and NO_3_^–^) was calculated using the equation: *R*_*v*_ = (T_1_-T_2_)/T_1_ × 100%, where *R*_*v*_ represents the nitrogen decrease efficiency, and T_1_ and T_2_ are the original and eventual contents of nitrogen in the system (mg L^–1^). Culture pH was measured by a pH meter.

The SPSS Statistics program (version 22) and Microsoft Excel 2010 were used for statistical analysis, and Origin 8.6 was used to produce the graphics.

## Results and discussion

### Impact of *nirBDs* on organic nitrogen transformation by strain Y-9

**FIGURE 1:**
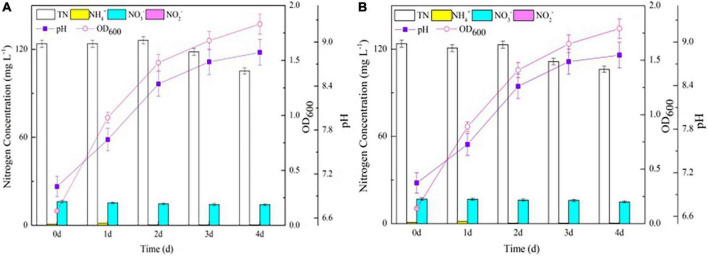
The growth curve and nitrogen transformation performance of strain Y-9 in organic nitrogen medium at 15^°^C. **(A)** The wild-type strain Y-9. **(B)** The △nirBD strain Y-9.

Both the wild-type and △*nirBD* strains grew vigorously in the OM and did not reach the stationary phase until 4 days (Figure 1), consistent with data from Fischer et al. (2012), who reported that strain *S. coelicolor* A3(2) (△*nirBD*) grew well on the plate containing casamino acids. The TN concentrations in the △*nirBD* and wild-type strain culture systems decreased gradually throughout the experiments and finally only dropped by 18.0 mg L^–1^ without NO_2_^–^ accumulation. This phenomenon suggested that strain Y-9 preferred to utilize organic nitrogen for cellular growth rather than converted it into gaseous nitrogen. Besides, variations in all of the measured nitrogen species within the culture medium of the wild-type and △*nirBD* strains were consistent across the entire incubation period. These results indicated that knocking out *nirBDs* did not affect organic nitrogen transformation by strain Y-9.

### Impact of *nirBDs* on ammonium transformation by strain Y-9

**FIGURE 2:**
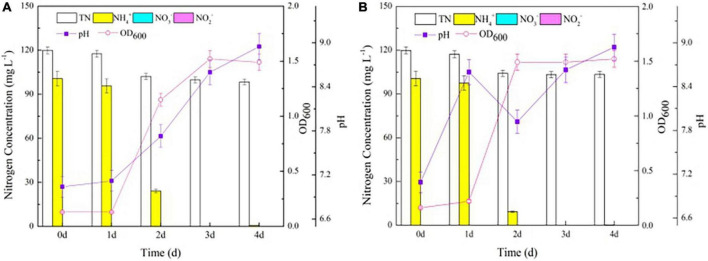
The growth curve and nitrogen transformation performance of strain Y-9 in nitrification medium at 15^°^C. **(A)** The wild-type strain Y-9. **(B)** The △nirBD strain Y-9.

To explore the effects of *nirBD* on the ammonium transformation process, the Y-9 and △*nirBD* strains were cultured in NM. Both strains grew vigorously (Figure 2), consistent with a previous study (Fischer et al., 2012), with the △*nirBD* strain of *S. coelicolor* growing similar to the wild-type strain on glucose minimal medium agar plates supplemented with NH_4_^+^ as the nitrogen source. These results illustrated that *nirBD* was not essential for the utilization of ammonium by strain Y-9. Intriguingly, the NH_4_^+^ removal efficiency by △*nirBD* strain (90.7%) was higher than that by the wild-type strain (76.1%) after 2 days of incubation (*P* < 0.05), which might be attributed to the stronger assimilation or ammonium oxidation ability of the △*nirBD* strain compared to the wild-type strain (Li et al., 2017; Jin et al., 2019).

The gas produced during the NH_4_^+^ removal process was N_2_O and not N_2_, according to the results of the GC test, consistent with our previous studies (Huang et al., 2019). Moreover, the δ^15^N/^14^N ratio of N_2_O in the △*nirBD* strain culture system (2.46) was lower than that in the wild-type strain culture system (3.63). Similarly, the decrease in TN in the △*nirBD* strain culture system (15.7 mg L^–1^) was lower than that in the wild-type strain culture system (21.5 mg L^–1^; *P* < 0.05; Figure 2). These results illustrated that knocking out *nirBDs* reduced the production of N_2_O, suggesting that the knockout accelerated ammonium assimilation instead of the ammonium oxidation by strain Y-9.

**FIGURE 3:**
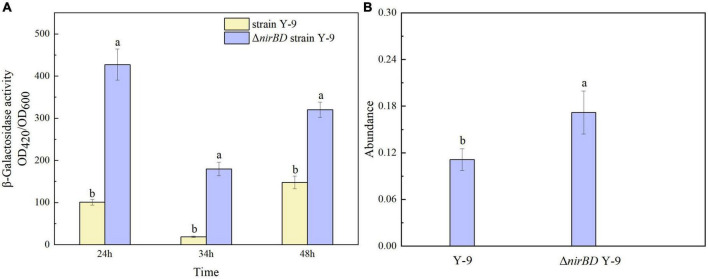
*GlnA* expression in strain Y-9 and △*nirBD* strain Y-9. **(A)** β-Galactosidase activity. **(B)** Relative expression abundance of *glnA*. The different lowercase letters above the bars indicate significant differences among treatments (*P* < 0.05).

Previous studies have proven that the glutamine synthetase encoded by *glnA* gene plays an important role in the ammonium assimilation process (Gupta et al., 2012; Van Heeswijk et al., 2013). In this study, *glnA* was found in strain Y-9 according to the results of the genome-wide scan. Considering that knocking out the *nirBDs* accelerated ammonium assimilation by strain Y-9, we speculated that the expression of *nirBDs* might influence the expression of g*lnA*. Thus, the expression of *glnA* in Y-9 and △*nirBD* strains was further detected. β-Galactosidase was utilized as a reporter to examine *glnA* promoter activity. The results showed that the β-galactosidase activity in the △*nirBD* strain was obviously higher than that in the wild-type strain (*P* < 0.05; Figure 3A). Moreover, qPCR results showed that the expression of *glnA* in the △*nirBD* strain was higher than that in wild-type strain Y-9 (Figure 3B). These findings suggested that knocking out *nirBDs* would promote the expression of *glnA*, accelerating ammonium assimilation.

### Impact of *nirBDs* on nitrate transformation by strain Y-9

**FIGURE 4:**
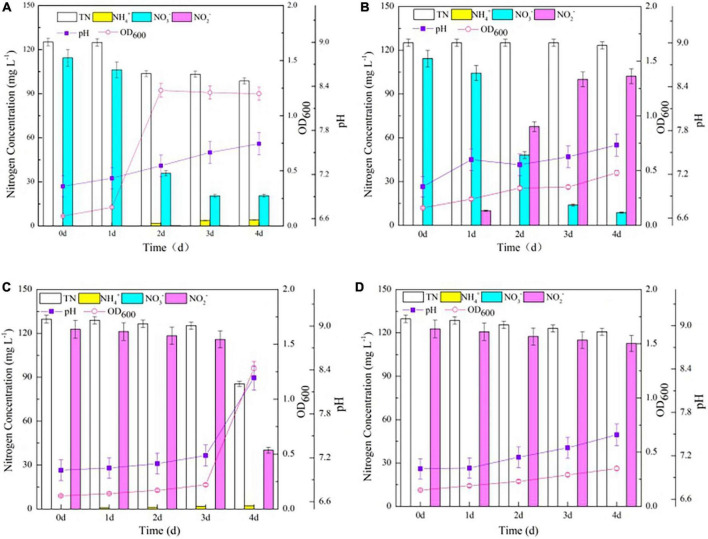
The growth curve and nitrogen transformation performance of strain Y-9 in denitrification medium at 15^°^C. **(A)** The wild-type strain Y-9 in DM-1. **(B)** The △nirBD strain Y-9 in DM-1. **(C)** The wild-type strain Y-9 in DM-2. **(D)** The △nirBD strain Y-9 in DM-2.

The OD_600_ of the wild-type strain increased significantly from 0.17 to 1.23, while the △*nirBD* strain exhibited a slower growth trend when culturing in SND-1 medium (*P* < 0.05; Figures 4A,B). These results are consistent with those observed when the two strains grew on agar plates supplemented with NO_3_^–^ as the sole nitrogen source but differed from those when strain NM7 (△*nirBD*) failed to grow under similar conditions (Fischer et al., 2012). The NirBD protein in strain Y-9 was previously shown to catalyze NO_2_^–^ reduction to NH_4_^+^ (Huang et al., 2020). Accordingly, a little amount of NO_2_^–^ was detected in our experiments, while NH_4_^+^ gradually increased during the cultivation of the wild type strain Y-9 (Figure 4A). In contrast, the accumulation of NO_2_^–^ was nearly equivalent to the decrease in NO_3_^–^, but NH_4_^+^ was undetectable during the entire NO_3_^–^ transformation process of the △*nirBD* strain (Figure 4B). These findings suggest that NO_2_^–^ converted from NO_3_^–^ could not be further reduced to NH_4_^+^ by strain Y-9 when *nirBDs* was knocked out, resulting in NO_2_^–^ accumulation in the cultures.

After cultivating the wild-type strain for 4 days, the decrease in NO_3_^–^ and TN reached 93.7 mg L^–1^ and 26.4 mg L^–1^, respectively. Moreover, culture pH increased over the whole cultivation period for the wild-type strain (Figure 4A). These dynamics were due to a small amount of NO_3_^–^ being removed by strain Y-9 via weak denitrification (Huang et al., 2020). The △*nirBD* strain achieved a total NO_3_^–^ reduction of 105.6 mg L^–1^, while TN was barely diminished after 4 d of incubation (Figure 4B). This finding could be because the knocking out of *nirBDs* resulted in NO_2_^–1^ accumulation in the medium, finally inhibiting the growth of strain Y-9 and its TN degrading ability.

### Impact of *nirBDs* on nitrite transformation by strain Y-9

Growth of the wild-type and △*nirBD* strains increased slowly during the initial 3 days of cultivation in the NO_2_^–^ -containing medium (Figures 4C,D). The probable reason for the slow growth was the high content of free nitrous acid (FNA, > 0.021 mg HNO_2_-N L^–1^ at 3 days) released due to the NO_2_^–^ inhibition in strain metabolism (Vadivelu et al., 2006). The wild-type strain grew quickly, with a concomitant considerable reduction of NO_2_^–^ and TN between days 3 and 4. After cultivation, the decrease in TN in suspension (44.2 mg L^–1^) was lower than the reduced amount of NO_2_^–^ in the supernatant (82.6 mg L^–1^; *P* < 0.05). Thus, some amount of NO_2_^–^ (44.2 mg L^–1^) might have been lost from the system through denitrification, while the remainder (38.4 mg L^–1^) could have been assimilated by the wild-type strain Y-9. NO_2_^–^ has well-documented toxicity to bacterial cells (Zemke et al., 2017), and strain Y-9 cannot directly absorb NO_2_^–^. Moreover, NH_4_^+^ accumulation was tracked throughout the NO_2_^–^ transformation process (Figure 4C). Therefore, it is possible that most of the NO_2_^–^ that had not been removed through denitrification could have been reduced to NH_4_^+^ through assimilation and DNRA by the wild-type strain Y-9, in accordance with our previous results (Huang et al., 2020). The △*nirBD* strain still grew at a slow rate after 3 days and could seldom remove NO_2_^–^. After 4 days of cultivation, NO_2_^–^ in the supernatant only decreased by 10.0 mg L^–1^, which was nearly equal to the decreased TN levels in the culture suspension (8.8 mg L^–1^), indicating that the △*nirBD* strain also conducted weak denitrification. Additionally, NH_4_^+^ was undetectable throughout the cultivation of the △*nirBD* strain (Figure 4D). Taken together, these results show that knocking out *nirBDs* does not allow noxious NO_2_^–^ to be reduced to NH_4_^+^, thereby inhibiting cellular growth and denitrification ability.

### Influence of *nirBDs* on nitrogen transformation of strain Y-9 in SND-1 medium

**FIGURE 5:**
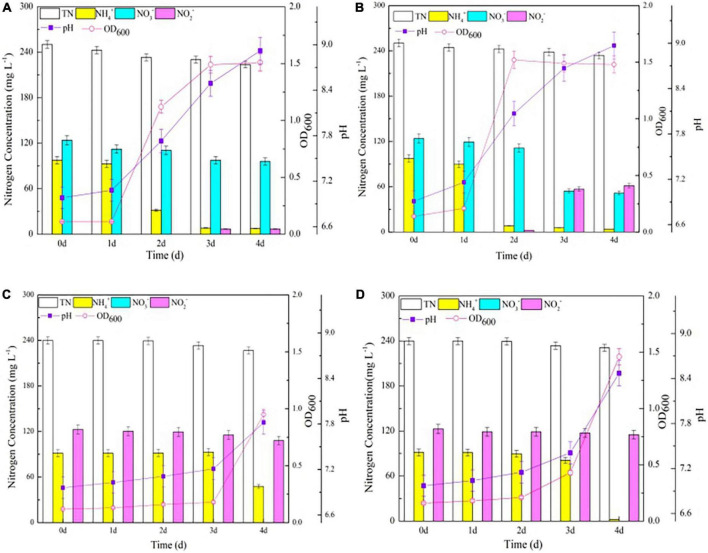
The growth curve and nitrogen transformation performance of strain Y-9 in simultaneous nitrification and denitrification medium. **(A)** The wild-type strain Y-9 in SND-1. **(B)** The △nirBD strain Y-9 in SND-1. **(C)** The wild-type strain Y-9 in SND-2. **(D)** The △nirBD strain Y-9 in SND-2).

Knocking out *nirBDs* accelerated the assimilation of NH_4_^+^ by strain Y-9 (Figure 2) and led to the near complete conversion of NO_3_^–^ into NO_2_^–^ while inhibiting the transformation of NO_2_^–^ (Figures 4B,D). We further evaluated the impact of *nirBDs* on nitrogen transformation when NH_4_^+^ and NO_3_^–^ coexisted in the medium. The wild-type and △*nirBD* strains grew vigorously after a 1-day lag phase and reached the stationary phase on days 3 and 2, respectively (Figures 5A,B). Concomitantly, the transformation of NH_4_^+^ by the △*nirBD* strain was faster than that of the wild-type strain (*P* < 0.05), consistent with the results when using NH_4_^+^ as the sole nitrogen source (Figure 2). These results were attributed to *nirBD* knockout that accelerated the assimilation of NH_4_^+^. The final NH_4_^+^ removal efficiency by the wild-type and △*nirBD* strains was 92.13 and 95.87%, respectively, which were both slightly lower than the NH_4_^+^ removal efficiency when incubating the two strains in NM (both approximately 100%) (Figure 2). Consequently, the existence of NO_3_^–^ led to little inhibition of NH_4_^+^ transformation. Moreover, the contents of TN in suspension both dropped down by approximately 20 mg L^–1^ in the two systems containing the wild type or △*nirBD* strain, consistent with the decrease in TN in suspension when NH_4_^+^ was used as the sole nitrogen source (Figure 2). Thus, no denitrification occurred in strain Y-9 when NH_4_^+^ and NO_3_^–^ coexisted in the system.

The decrease in NO_3_^–^ content in suspension reached only 26.4 mg L^–1^, and the NO_2_^–^ was undetected after 4 days of wild type strain cultivation, indicating that strain Y-9 utilized NH_4_^+^ preferentially when NH_4_^+^ and NO_3_^–^ coexisted in the medium. Similar results were observed by Xu et al. (2017), who used 200 mg L^–1^ of NH_4_^+^ and NO_3_^–^ to cultivate strain Y-9. Yang J. R. et al. (2019) and Zhang et al. (2022) also reported that *Acinetobacter* sp. JR1 and *P. taiwanensis* EN-F2 preferred to remove NH_4_^+^ from a medium containing both NH_4_^+^ and NO_3_^–^. Nevertheless, the contents of NO_3_^–^ in the △*nirBD* strain cultures dropped by 72.3 mg L^–1^ and the accumulation of NO_2_^–^ reached 61.5 mg L^–1^ at the end of the experiment (Figure 5B). The above results combined with variation in NO_3_^–^ and NO_2_^–^ concentrations when cultivating the △*nirBD* strain in DM-1 medium (Figure 4B) suggested that *nirBDs* reduced the NO_2_^–^ resulting from NO_3_^–^ respiration to NH_4_^+^, and the denitrification ability of the △*nirBD* strain was weak. In addition, when using NH_4_^+^ or NO_3_^–^ as the sole nitrogen source, the pH increased over the entire incubation process of the wild-type strain Y-9 but fluctuated during △*nirBD* strain growth (Figures 2, 4A,B). Intriguingly, the pH increased during the entire cultivation period of the wild-type and △*nirBD* strains when NH_4_^+^ and NO_3_^–^ were both available (Figures 5A,B). Thus, the coexistence of NH_4_^+^ and NO_3_^–^ might counteract the effects of *nirBDs* knockout with regard to culture pH. Nevertheless, these dynamics require further investigation.

### Influence of *nirBDs* on the nitrogen transformation of strain Y-9 in the SND-2 medium

When NH_4_^+^ and NO_2_^–^ coexisted in the medium, the wild-type and △*nirBD* strains both barely grew within the first 2 days. Nevertheless, the △*nirBD* strain exhibited higher growth than the wild type strain over the entire cultivation period (*P* < 0.05; Figures 5C,D), owing to the acceleration of NH_4_^+^ assimilation by strain Y-9, due to *nirBDs* knockout (Figure 2). The NH_4_^+^ removal rate by the △*nirBD* strain (97.5%) was considerably higher than that of the wild-type strain (47.7%), but the decrease in TN in the △*nirBD* strain culture system (9.1 mg L^–1^) was lower than that in the wild-type strain culture system (13.2 mg L^–1^; *P* < 0.05). This finding could be attributed to the denitrification that was inhibited when *nirBDs* were knocked out (Figures 4A,B). The decrease in NO_2_^–^ in the wild-type strain culture system was consistently greater than that of the △*nirBD* strain culture system over the entire incubation period (*P* < 0.05), which might have been due to the stronger denitrification rate of the wild-type strain compared to the △*nirBD* strain. However, the high concentration of NO_2_^–^ in the system could still inhibit the utilization of NH_4_^+^ by the wild-type strain, with residual NH_4_^+^ levels reaching 47.8 mg L^–1^ at the end of the experiment (Figure 5C), consistent with the reports that the addition of NO_2_^–^ had a negative impact on ammonium removal of bacterium (Yang et al., 2012; Zhang et al., 2015, 2022). A noteworthy observation is that NO_2_^–^ had no impact on the ammonium efficiency of the △*nirBD* strain, which reached 97.5% (Figure 5D), possibly due to *nirBDs* knockout leading to increased NH_4_^+^ assimilation and strain growth, thereby enhancing the tolerance of the strain to the toxic NO_2_^–^.

## Conclusion

*nirBDs*, which conventionally encode an NADH nitrite reductase, also influence the ammonium transformation of *P. putida* Y-9. Knocking out *nirBDs* accelerated the ammonium assimilation and inhibited the emission of the greenhouse gas N_2_O, thus alleviating the toxicity of nitrite in an ammonium and nitrite system.

## Data availability statement

The datasets presented in this study can be found in online repositories. The names of the repository/repositories and accession number(s) can be found below: GenBank, KP410740.

## Author contributions

XH: visualization, investigation, data curation, writing—original draft preparation, reviewing, and funding acquisition. YL: investigation, data curation, and formal analysis. LL: investigation and data curation. DX: project administration and supervision. ZL: conceptualization, methodology, and writing—reviewing and editing. All authors contributed to the article and approved the submitted version.

## Supplementary material

Supplementary Figure 1 *nirBD* deletion mutations detection (Lanes 1–6: deletion mutant; Lane 8: wild-type strain Y-9; Lane 9: DL5000 DNA Marker).

## References

[B1] Ai G. M. Zheng H. Y. Zhang M. Liu Z. P. (2011). Isotopic confirmation of occurrence of microbial denitrification based on N_2_ and N_2_O production monitored by gas chromatography/isotope ratio mass spectrometry and gas chromatography/mass spectrometry. *Chin. J. Anal. Chem.* 39 1141–1146. 10.1016/S1872-2040(10)60460-4

[B2] Burkovski A. (2003). Ammonium assimilation and nitrogen control in *Corynebacterium glutamicum* and its relatives: An example for new regulatory mechanisms in actinomycetes. *FEMS Microbiol. Rev.* 27 617–628. 14638415 10.1016/S0168-6445(03)00067-6

[B3] Canfield D. E. Glazer A. N. Falkowski P. G. (2010). The evolution and future of Earth’s nitrogen cycle. *Science* 330 192–196. 10.1126/science.1186120 20929768

[B4] Chen J. L. Xu J. Zhang S. N. Liu F. Peng J. W. Peng Y. X. (2021). Nitrogen removal characteristics of a novel heterotrophic nitrification and aerobic denitrification bacteria, *Alcaligenes faecalis* strain WT14. *J. Environ. Manage.* 282 111961. 10.1016/j.jenvman.2021.111961 33465711

[B5] Chen J. Gu S. Y. Hao H. H. Chen J. M. (2016). Characteristics and metabolic pathway of *Alcaligenes* sp. TB for simultaneous heterotrophic nitrification-aerobic denitrification. *Appl. Microbiol. Biot.* 100 9787–9794. 10.1007/s00253-016-7840-x 27678119

[B6] Fischer M. Schmidt C. Falke D. Sawers R. G. (2012). Terminal reduction reactions of nitrate and sulfate assimilation in *Streptomyces coelicolor* A3 (2): Identification of genes encoding nitrite and sulfite reductases. *Res. Microbiol.* 163 340–348. 10.1016/j.resmic.2012.05.004 22659143

[B7] Gennis R. B. Stewart V. (1996). “Respiration,” in *Escherichia coli and Salmonella-Cellular and Molecular Biology*, eds Neidhardt F. C. Curtiss R. III Ingraham J. L. Lin E. C. C. Low K. B. Magasanik B. (Washington, DC: ASM Press).

[B8] Gupta N. Gupta A. K. Gaur V. S. Kumar A. (2012). Relationship of nitrogen use efficiency with the activities of enzymes involved in nitrogen uptake and assimilation of finger millet genotypes grown under different nitrogen inputs. *Sci. World J.* 2012:625731. 10.1100/2012/625731 22919342 PMC3415157

[B9] Huang X. J. Jiang D. H. Ni J. P. Xie D. T. Li Z. L. (2021a). Removal of ammonium and nitrate by the hypothermia bacterium *Pseudomonas putida* Y-9 mainly through assimilation. *Environ. Technol. Innov.* 22:101458.

[B10] Huang X. J. Tie W. Z. Xie D. T. Jiang D. H. Li Z. L. (2021b). Certain environmental conditions maximize ammonium accumulation and minimize nitrogen loss during nitrate reduction process by *Pseudomonas putida* Y-9. *Front. Microbiol.* 12:764241. 10.3389/fmicb.2021.764241 34966364 PMC8710668

[B11] Huang X. J. Weisene C. G. Ni J. P. He B. H. Xie D. T. Li Z. L. (2020). Nitrate assimilation, dissimilatory nitrate reduction to ammonium, and denitrification coexist in *Pseudomonas putida* Y-9 under aerobic conditions. *Bioresour. Technol.* 312:123597. 10.1016/j.biortech.2020.123597 32506044

[B12] Huang X. J. Xu Y. He T. X. Jia H. J. Feng M. Xiang S. D. (2019). Ammonium transformed into nitrous oxide via nitric oxide by *Pseudomonas putida* Y-9 under aerobic conditions without hydroxylamine as intermediate. *Bioresour. Technol.* 277 87–93. 10.1016/j.biortech.2019.01.040 30660065

[B13] Jackson R. H. Cornish-Bowden A. Cole J. A. (1981). Prosthetic groups of the NADH-dependent nitrite reductase from *Escherichia coli* K12. *Biochem J.* 193 861–867. 10.1042/bj1930861 7030314 PMC1162678

[B14] Jin P. Chen Y. Y. Yao R. Zheng Z. W. Du Q. Z. (2019). New insight into the nitrogen metabolism of simultaneous heterotrophic nitrification-aerobic denitrification bacterium in mRNA expression. *J. Hazard Mater.* 371, 295–303. 10.1016/j.jhazmat.2019.03.023 30856440

[B15] Jin R. F. Liu T. Q. Liu G. F. Zhou J. T. Huang J. Y. Wang A. J. (2015). Simultaneous heterotrophic nitrification and aerobic denitrification by the marine origin bacterium *Pseudomonas* sp. ADN-42. *Appl. Biochem. Biotech.* 175 2000–2011. 10.1007/s12010-014-1406-0 25432342

[B16] Kuypers M. M. M. Marchant H. K. Kartal B. (2018). The microbial nitrogen-cycling network. *Nat. Rev. Microbiol.* 16 263–276. 2018.10.060 29398704 10.1038/nrmicro.2018.9

[B17] Lei X. Jia Y. T. Chen Y. C. Hu Y. Y. (2019). Simultaneous nitrification and denitrification without nitrite accumulation by a novel isolated *Ochrobactrum anthropic* LJ81. *Bioresour. Technol.* 272 442–450. 10.1016/j.biortech 30388582

[B18] Lei Y. Wang Y. Q. Liu H. J. Xi C. W. Song L. Y. (2016). A novel heterotrophic nitrifying and aerobic denitrifying bacterium, *Zobellella taiwanensis* DN-7, can remove high-strength ammonium. *Appl. Microbiol. Biot.* 100 4219–4229. 10.1007/s00253-016-7290-5 26762390

[B19] Li Y. T. Wang Y. R. Fu L. Gao Y. Z. Zhao H. X. Zhou W. Z. (2017). Aerobic-heterotrophic nitrogen removal through nitrate reduction and ammonium assimilation by marine bacterium Vibrio sp. Y1-5. *Bioresour. Technol.* 230, 103–111. 10.1016/j.biortech.2017.01.049 28167356

[B20] Lin J. T. Stewart V. (1997). Nitrate assimilation by bacteria. *Adv. Microb. Physiol.* 39 1–30. 10.1016/s0065-2911(08)60014-4 9328645

[B21] Macdonald H. Pope N. R. Cole J. A. (1985). Isolation, characterization and complementation analysis of *nirB* mutants of *Escherichia coli* deficient only in NADH-dependent nitrite reductase activity. *Microbiology* 131 771–2782. 10.1099/00221287-131-10-2771 3906030

[B22] Malm S. Tiffert Y. Micklinghoff J. Schultze S. Joost I. Weber I. (2009). The roles of the nitrate reductase NarGHJI, the nitrite reductase NirBD and the response regulator GlnR in nitrate assimilation of *Mycobacterium tuberculosis*. *Microbiology* 155 1332–1339. 10.1099/mic.0.023275-0 19332834

[B23] Miller J. H. (1972). *Experiments in Molecular Genetics.* Cold Spring Harbor, NY: Cold spring harbor laboratory.

[B24] Muro-Pastor M. I. Reyes J. C. Florencio F. J. (2005). Ammonium assimilation in cyanobacteria. *Photosynth. Res.* 83 135–150. 10.1007/s11120-004-2082-7 16143848

[B25] State Environmental Protection Administration of China (2002). *Water and Wastewater Analysis Methods.* Beijing: China Environmental Science Press, 132–286.

[B26] Tiffert Y. Supra P. Wurm R. Wohlleben W. Wagner R. Reuther J. (2008). The *Streptomyces coelicolor* GlnR regulon: Identification of new GlnR targets and evidence for a central role of GlnR in nitrogen metabolism in actinomycetes. *Mol. Microbiol.* 67 861–880. 10.1016/j.biortech.2014.08.058 18179599

[B27] Vadivelu V. M. Keller J. Yuan Z. (2006). Effect of free ammonia and free nitrous acid concentration on the anabolic and catabolic processes of an enriched *Nitrosomonas* culture. *Biotechnol. Bioeng.* 95 830–839. 10.1002/bit.21018 16960893

[B28] Van Heeswijk W. C. Westerhoff H. V. Boogerd F. C. (2013). Nitrogen assimilation in *Escherichia coli*: Putting molecular data into a systems perspective. *Microbiol. Mol. Biol. R.* 77 628–695. 10.1128/MMBR.00025-13 24296575 PMC3973380

[B29] Xia L. Li X. M. Fan W. H. Wang J. L. (2020). Heterotrophic nitrification and aerobic denitrification by a novel *Acinetobacter* sp. ND7 isolated from municipal activated sludge. *Bioresour. Technol.* 301:122749. 10.1016/j.biortech.2020.122749 31951959

[B30] Xu L. Gu G. Q. Wei C. Gao L. J. Wu X. H. Zhang L. Q. (2018). The outer membrane protein OprF and the sigma factor SigX regulate antibiotic production in *Pseudomonas fluorescens* 2P24. *Microbiol. Res.* 206 159–167. 10.1016/j.micres.2017.10.006 29146252

[B31] Xu Y. He T. X. Li Z. L. Ye Q. Chen Y. L. Xie E. Y. (2017). Nitrogen removal characteristics of *Pseudomonas putida* Y-9 capable of heterotrophic nitrification and aerobic denitrification at low temperature. *Biomed. Res. Int.* 2017:1429018. 10.1155/2017/1429018 28293626 PMC5331289

[B32] Yan Q. Wu X. G. Wei H. L. Wang H. M. Zhang L. Q. (2009). Differential control of the PcoI/PcoR quorum-sensing system in *Pseudomonas fluorescens* 2P24 by sigma factor RpoS and the GacS/GacA two-component regulatory system. *Microbiol. Res.* 164 18–26. 10.1016/j.micres.2008.02.001 18395434

[B33] Yang J. R. Wang Y. Chen H. Lyu Y. K. (2019). Ammonium removal characteristics of an acid-resistant bacterium *Acinetobacter* sp. JR1 from pharmaceutical wastewater capable of heterotrophic nitrification-aerobic denitrification. *Bioresour. Technol.* 274 56–64. 10.1016/j.biortech.2018.10.052 30500764

[B34] Yang L. Wang X. H. Cui S. Ren Y. X. Yu J. Chen N. (2019). Simultaneous removal of nitrogen and phosphorous by heterotrophic nitrification-aerobic denitrification of a metal resistant bacterium *Pseudomonas putida* strain NP. *Bioresour. Technol.* 285 121360. 10.1016/j.biortech.2019.121360 31015182

[B35] Yang X. P. Wang S. M. Zhou L. X. (2012). Effect of carbon source, C/N ratio, nitrate and dissolved oxygen concentration on nitrite and ammonium production from denitrification process by *Pseudomonas stutzeri* D6. *Bioresour. Technol.* 104 65–72. 10.1016/j.biortech.2011.10.026 22074905

[B36] Ye J. Zhao B. An Q. Huang Y. S. (2016). Nitrogen removal by *Providencia rettgeri* strain YL with heterotrophic nitrification and aerobic denitrification. *Environ. Technol.* 37 2206–2213. 10.1080/09593330.2016.1146338 26824874

[B37] Yukioka Y. Tanahashi T. Shida K. Oguchi H. Ogawa S. Saito C. (2017). A role of nitrite reductase (NirBD) for NO homeostatic regulation in *Streptomyces coelicolor*A3(2). *FEMS Microbiol. Lett.* 364:fnw241. 10.1093/femsle/fnw241 27797866

[B38] Zemke A. C. Kocak B. R. Bomberger J. M. (2017). Sodium nitrite inhibits killing of *Pseudomonas aeruginosa* biofilms by ciprofloxacin. *Antimicrob. Agents Chemother.* 61:e00448-16. 10.1128/aac.00448-16 27799207 PMC5192113

[B39] Zhang M. M. He T. X. Chen M. P. Wu Q. F. (2022). Ammonium and hydroxylamine can be preferentially removed during simultaneous nitrification and denitrification by *Pseudomonas taiwanensis* EN-F2. *Bioresour. Technol.* 350:1269126. 10.1016/j.biortech.2022.126912 35231598

[B40] Zhang M. X. Li A. Z. Yao Q. Wu Q. P. Zhu H. H. (2020). Nitrogen removal characteristics of a versatile heterotrophic nitrifying-aerobic denitrifying bacterium, *Pseudomonas bauzanensis* DN13-1, isolated from deep-sea sediment. *Bioresour. Technol.* 305:122626. 10.1016/j.biortech.2019.122626 32143020

[B41] Zhang Y. Shi Z. Chen M. X. Dong X. Y. Zhou J. T. (2015). Evaluation of simultaneous nitrification and denitrification under controlled conditions by an aerobic denitrifican culture. *Bioresour. Technol.* 175 602–605. 10.1016/j.biortech.2014.10.016 25455090

