# Supplementary material for: The nitrite reductase encoded by nirBDs in Pseudomonas putida Y-9 influences ammonium transformation
Source: Front Microbiol. 2022 Oct 12;13:982674. doi: 10.3389/fmicb.2022.982674 (PMC9597696; doi:10.3389/fmicb.2022.982674)
Supplement: Supplementary file 2 [file Table_1.docx]

| Table S1 Sequences of primers used for *nirBD* gene knockout | |  |
| --- | --- | --- |
| Primers | Sequences | |
| NirBD-MF1-1 | TCTATGTGCAGCAGCGCATG | |
| NirBD-MR1-1 | GATGACCAGTCGCTCTCGTTG | |
| NirBD-MF2-1 | CTGCACCTGATCCCAACTGTC | |
| NirBD-MR2-1 | CGAGGTTCATTGCAGCAAGCT | |
| NirBD-TF | TAACTGGCTGTTCTTTGGCG | |
| NirBD-TR | TTGCAGCAGGTCTTCGAGTTC | |
