# Supplementary figures and images for: The nitrite reductase encoded by nirBDs in Pseudomonas putida Y-9 influences ammonium transformation
Source: Front Microbiol. 2022 Oct 12;13:982674. doi: 10.3389/fmicb.2022.982674 (PMC9597696; doi:10.3389/fmicb.2022.982674)

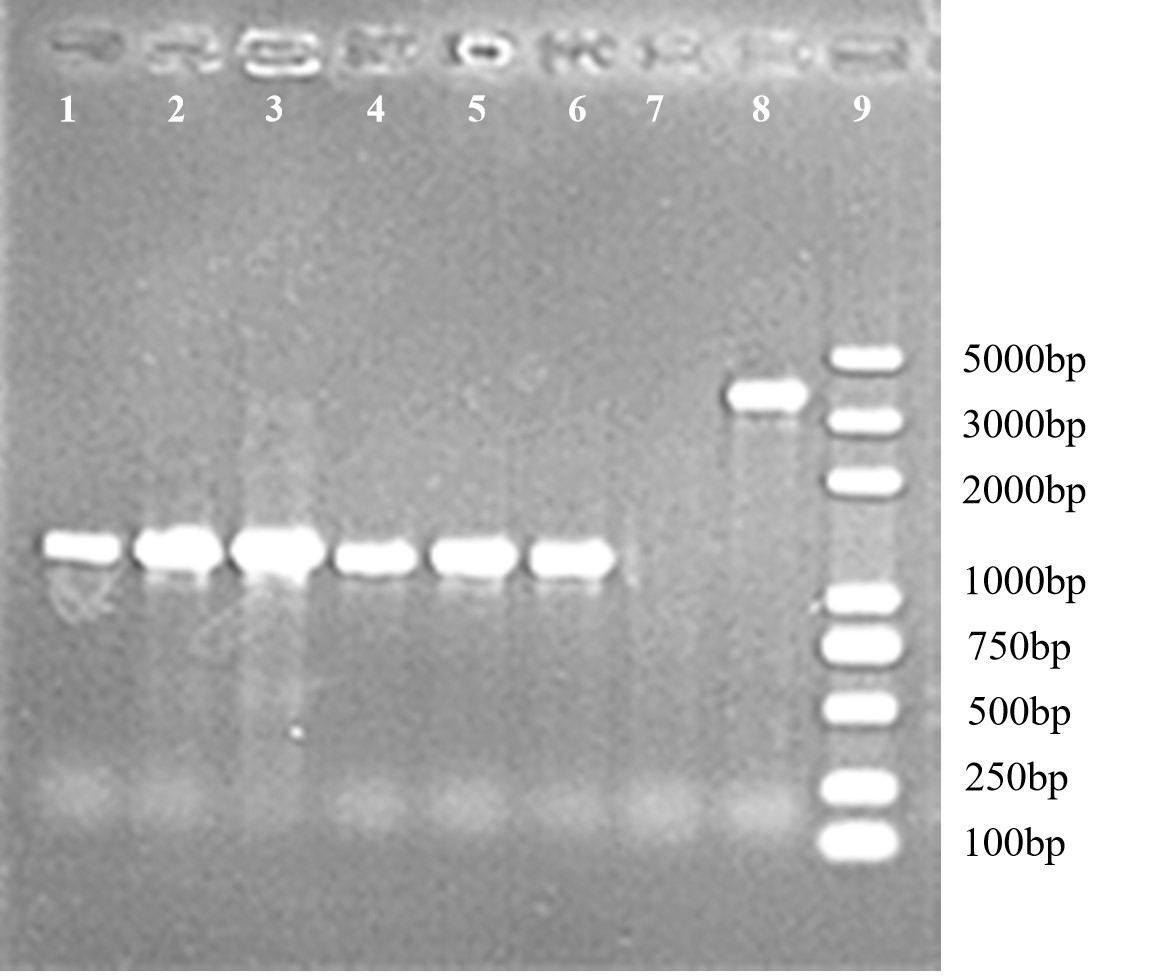

Supplement: Supplementary Figure 1 — nirBD deletion mutations detection (Lanes 1–6: deletion mutant; Lane 8: wild-type strain Y-9; Lane 9: DL5000 DNA Marker). [file Image_1.JPEG]
